# Performance evaluation of electrosurgical pencils: insights into surgical smoke mitigation and thermal damage reduction

**DOI:** 10.1097/JS9.0000000000005093

**Published:** 2026-03-17

**Authors:** Kihyun Sang, Yoon Kong, Ja Kyung Lee, Hee Young Na, Hyeong Won Yu, June Young Choi

**Affiliations:** a Department of Surgery, Seoul National University Bundang Hospital, Seongnam-si, Gyeonggi-do, Republic of Korea; b Department of Pathology, Seoul National University Bundang Hospital, Seongnam, Republic of Korea; c Department of Pathology, Seoul National University College of Medicine, Seoul, Republic of Korea; d Department of Surgery, Seoul National University College of Medicine, Seoul, Republic of Korea

**Keywords:** electrosurgical pencil (EPS), hemostasis, surgical smoke

## Abstract

**Background:**

Electrosurgical pencils (ESPs) are essential in modern surgery; however, concerns remain regarding their exposure to surgical smoke and thermal tissue damage. This study quantitatively evaluated four ESPs with a particular focus on two critical aspects: surgical smoke generation and minimization of thermal damage to the surrounding tissues.

**Methods:**

Full-thickness incisions were made on the porcine dorsal skin using six instruments: scalpel, uncoated Bovie, coated Bovie, PlasmaBlade, and Duoblade (with and without suction). Surgical smoke was measured by particle counting, blood loss was quantified, and histological analyses (hematoxylin and eosin, Masson’s trichrome, CD3, and α-smooth muscle actin staining) were performed to assess the wound scar width and densities of CD3-positive lymphocytes and myofibroblasts.

**Results:**

Duoblades, especially those with suction, consistently produced the lowest level of surgical smoke, reducing particle counts by up to 96.1% compared to the uncoated Bovie. It also achieved the greatest reduction in blood loss (93.3% less than that of the scalpel) and resulted in the narrowest scar width among the ESPs. The wound scar width and densities of CD3-positive lymphocytes and myofibroblasts were reduced more rapidly in Duoblade-incised wounds than in those with other ESPs.

**Discussion:**

Device design and energy delivery significantly affect the surgical smoke and tissue outcomes. Duoblade dielectric heating and integrated suction provide superior environmental safety and wound healing, while maintaining excellent hemostasis. The Duoblade has a suction function that reduces the surgical smoke.

## Introduction

Electrosurgical pencils (ESPs) were first used in surgery in 1926 through the collaboration between William T. Bovie and Harvey Cushing, establishing their indispensable role in modern surgical practices^[^1^]^. Over the past century, continuous efforts have aimed to prevent ESP-associated complications. Technological advancements, such as insulation and low-temperature dissection, have been developed to minimize unnecessary thermal damage. The PlasmaBlade (Medtronic), which delivers low-temperature cauterization (approximately 40–100°C) through waveform modulation, marked the beginning of efforts to reduce thermal exposure in electrosurgery^[^2^]^. PlasmaBlade has enabled more focused research on temperature-dependent wound healing and supported practical discussions regarding the clinical feasibility of low-temperature electrosurgical devices^[^3–6^]^.

HIGHLIGHTS

- This study is a quantitative evaluation of various electrosurgical pencils, focusing on surgical smoke generation and minimizing thermal tissue damage using a porcine model.
- The core findings demonstrate that the Duoblade device, especially when using integrated suction, significantly reduces surgical smoke exposure and achieves superior hemostasis while minimizing tissue inflammation and fibrosis compared to conventional devices.
- These results highlight the potential of Duoblade to enhance environmental safety in the operating room and improve post-surgical wound healing, suggesting a meaningful advance in both occupational safety and patient outcomes.

Although minimizing thermal damage remains a primary goal, increasing interest has focused on the health effects of surgical smoke^[^7–12^]^. Studies have revealed that surgical smoke contains toxic chemicals, infectious particles, and fine particulate matter, posing risks, such as respiratory illnesses and potential carcinogenicity, to operating room personnel. Professional bodies advocate for robust measures to mitigate these health risks, including local smoke evacuation systems near the ESP tip, which have proven effective across various tissue types^[^13^]^.

The Duoblade (Dielectric Ultra-focused Oscillatory Blade, CRESEN, Inc.) was introduced as a novel low-temperature dissection ESP to reduce thermal damage and surgical smoke. Unlike conventional ESPs, which rely on Joule heating, the Duoblade employs a dielectric heating mechanism that uses a high-frequency electric field to induce the frictional heating of polar molecules (primarily water) within the tissue. If water molecules vaporize, the heating effect ceases naturally, resulting in a self-limiting temperature profile around the boiling point of water (approximately 100°C). The Duoblade features a fully insulated electrode tip, using dielectric heating as the primary mechanism for tissue dissection and coagulation. Preclinical studies have highlighted the Duoblade’s low-temperature operation and potential to minimize necrosis in tissues adjacent to incisions because of its fully insulated electrode design and dielectric heating mechanism^[^14^]^.

Building on the promise of low-temperature electrosurgery, this study aimed to provide a comparative analysis of four ESPs: two conventional high-temperature ESPs [uncoated Bovie (REF130309A, CONMED) and coated Bovie (SEP6000, Covidien)] and two low-temperature dissection ESPs [PlasmaBlade (PS210-030S, Medtronic) and Duoblade (DB1SEP, Cresen)]. This study focused on three critical aspects of the surgical performance and outcomes in a porcine model. To quantify and compare the acute surgical efficacy, intraoperative blood loss associated with each ESP was measured. The safety profile was evaluated by quantifying the concentration of fine particulate matter (surgical smoke) generated during the incision. To evaluate the lasting impact of different energy profiles, postoperative tissue healing was monitored for 4 weeks. Comprehensive histological analyses, including hematoxylin and eosin (H&E), Masson’s trichrome (MT), CD3, and α-SMA staining, were performed to evaluate necrosis, inflammation, and fibrosis at the surgical site.

By analyzing three distinct and critical outcome measures, such as blood loss, surgical smoke generation, and long-term histological healing, this study provides robust comparative evidence of how engineering differences in electrosurgical devices translate into measurable differences in immediate surgical performance and biological consequences.

The authors confirm that no artificial intelligence tools or large language models were used in the drafting, writing, or editing of this manuscript^[^15^]^.

## Methods

### Operation protocol

This study was designed and reported according to the ARRIVE (Animal Research: Reporting of *In Vivo* Experiments) guidelines to ensure transparency and reproducibility^[^16–18^]^. The pigs used in this experiment were 40 kg male (Yorkshire × Landrace × Duroc) pigs (*Sus scrofa domestica*), which underwent an acclimatization period in a constant housing environment for 1 week before the experiment. The housing facility was maintained at a temperature of 20 ± 2°C and a humidity of 50 ± 10%. Lighting was performed for 12 h/day, and the remaining 12 h was allowed to rest in the dark. Water was provided *ad libitum*, and the pigs were fed once daily at 2% of their body weight. To maintain cleanliness, the floor was cleaned daily, and regular disinfection (walls, floor, and ceiling) was performed according to a disinfection program. A sufficient sample size for the main test (surgical smoke) was secured for each ESP (*n* = 16). Consequently, if the effect size (*η*^2^) was moderate or higher according to Cohen’s guidelines, in which an *η*^2^ value of >0.14 is considered a large effect, the statistical power may be adequate^[^19^]^.

**Figure 1.**
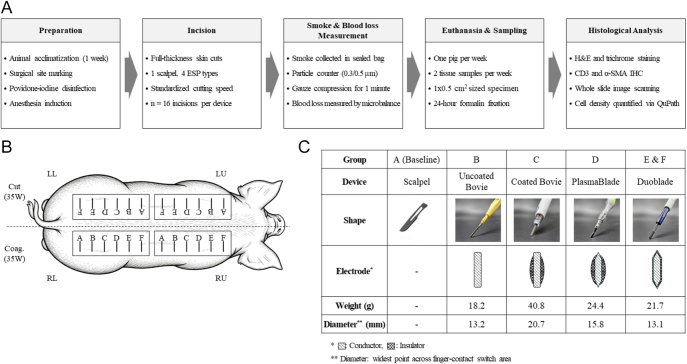
Overall experimental design and workflow for assessing electrosurgical incision effects in a porcine model. (a) Experimental workflow, (b) incision sites, and (c) devices used.

Following general anesthesia, the surgical sites were marked and disinfected with povidone-iodine. The dorsal skin of each pig was divided into quadrants, and six instruments, namely, scalpel, uncoated Bovie, coated Bovie, PlasmaBlade, Duoblade without suction, and Duoblade with suction, were used to create full-thickness incisions, each 5 cm in length and spaced 3 cm apart. Pre-testing confirmed that Duoblade’s integrated suction operated within the typical efficiency range of 70–80%, which is consistent with other commercial ESPs, and was included in the experiment. Two zones on the left side were incised using the cut mode (power: 35 W), while two zones on the right side were incised using the coagulation mode (power: 35 W). During each incision, surgical smoke was collected and blood loss was measured (Fig. 1).

### Euthanasia protocol

One pig was euthanized every week over a 4-week period, yielding four sets of samples. The histological samples were processed to generate pathological slides.

### Assessment of surgical smoke

Surgical smoke was collected in a sealed antistatic-treated plastic bag positioned 3 cm away from the incision site for 10 s during the incision procedure. Anti-static treatment of the plastic bag was employed to prevent uneven distribution of particle density during collection. An antistatic plastic bag with a maximum volume capacity of approximately 200 L was used for smoke collection. A high-capacity air pump (rated at 600 liters per minute (LPM)) was used to rapidly transfer surgical smoke into the collection bag. Ventilation was performed to prevent cross-contamination between quadrants, and the procedure was resumed only after the contamination level fell below the baseline. After collection, the bag was sealed and stabilized for 10 min to eliminate internal air currents and ensure homogenous particle distribution. Quantification was performed by connecting the inlet of a light-scattering airborne particle counter (TSI AeroTrak 9303) to the bag. The device was operated at a sampling flow rate of 2.83 LPM, allowing for accurate and consistent particle counting. The collected smoke was analyzed using the particle counter to determine the densities of particles measuring 0.3 and 0.5 μm because surgical smoke generated from skin tissues predominantly falls within the particle size range of 0.1–10 μm^[^20^]^. Additionally, according to studies comparing surgical smoke generated by electrosurgery, lasers, and ultrasound knives, the sizes of the particles in electrosurgical smoke are distributed between 0.07 and 0.42 µm^[^9^]^. Considering that particles in the micrometer range are filterable by standard surgical masks, this study focused on quantifying ultrafine particles that may pose greater health risks to medical personnel in real surgical environments.

### Assessment of blood loss

Modern electrosurgical units are designed to regulate frequency, which helps minimize risks such as burns, nerve stimulation, and muscle contractions. Additionally, variations in voltage and current waveforms influence the extent of heat generation and vaporized tissue by-products^[^21^]^. Given that the differences in the frequency and electromagnetic field design among the devices under investigation may lead to variations in tissue healing and blood loss, a series of analyses were conducted to examine these effects.

After each incision, gauze was applied with pressure for 1 min to collect blood loss. Immediately after this period, the gauze was weighed using a high-precision microbalance (CAS MWII-300H; CAS, Korea) with a precision of 0.01 g, and net blood loss was recorded by subtracting the pre-measured weight of the gauze. To ensure consistency, the same type of gauze was used throughout the experiment, assuming a uniform initial weight as in similar studies^[^22–24^]^. All procedures were performed under sterile conditions. All incision and hemostasis procedures were performed by a single experienced surgeon after sufficient practice to minimize interoperator variability. The cutting speed was strictly controlled at 1 cm/s and the incision depth was standardized to approximately 5 mm to ensure penetration of the skin, dermis, and subcutaneous layers. To avoid interference with blood loss measurement, gauze compression during hemostasis was applied with a controlled pressure of 150 g (±10 %). Sixteen data points were collected per ESP across the four pigs.

### Histological analysis

For each porcine model, the left quadrants were incised using the cut mode, whereas the right quadrants were incised using the coagulation mode. One sample from the cut mode quadrant and one from the coagulation mode quadrant were collected from each animal. A 1 × 0.5-cm specimen, including the incision site, was excised, fixed in 10% buffered formalin for 24 h, and embedded in paraffin. Formalin-fixed paraffin-embedded blocks were sectioned at a thickness of 4 µm and stained with H&E and MT. Immunohistochemical (IHC) staining was performed using antibodies against CD3 (Cell Marque, RTU, polyclonal) and α-smooth muscle actin (SMA; Cell Marque, RTU, 1A4).

The thermal damage induced by ESPs has been widely studied in the context of tissue injury, inflammation, and fibrosis. H&E staining allows for the assessment of coagulative necrosis, cellular integrity, and overall tissue architecture following thermal injury^[^2,25,26^]^. MT staining was performed to evaluate collagen deposition, extracellular matrix remodeling, and fibrotic changes following ESP-induced tissue injury^[^27^]^. Excessive collagen accumulation and extracellular matrix remodeling contribute to fibrotic tissue responses, which can be influenced by the electrosurgical settings and energy profiles^[^28^]^.

Additionally, IHC markers such as CD3 (T-cell marker) and α-SMA (myofibroblast marker) have been validated as reliable indicators of the inflammatory response and fibrosis, respectively, in thermally injured tissues^[^29,30^]^. Based on this evidence, H&E staining was employed to evaluate general tissue morphology and necrosis, while IHC staining of CD3 and α-SMA was performed to quantify immune cell infiltration and fibrotic tissue remodeling at the incision site. The combination of H&E, MT, and IHC (CD3 and α-SMA) staining provided a comprehensive histological assessment, allowing a detailed investigation of necrosis, fibrosis, and inflammatory cell infiltration at the incision site.

All slides were digitally scanned using an Aperio GT450 scanner (Leica Biosystems, Nussloch, Germany) and interpreted by a board-certified pathologist (H.Y.N.) in a blinded manner. The wound scar width was evaluated using MT-stained slides. In CD3- and α-SMA-stained slides, a pathologist carefully selected one representative region per case, encompassing the area with the most prominent inflammatory or fibrotic changes after thorough review of the entire lesion. To minimize bias related to the region size, the number of positive cells per mm^2^ (the total number of positive cells within a specific area divided by the area size) was used for comparison. This value was automatically computed using the open-source QuPath software^[^31^]^.

## Results

### Surgical smoke

Surgical smoke generation varied significantly according to ESP type and incision mode (cut vs. coagulation). The density was measured for 0.3- and 0.5-µm particles (particles/cubic foot). Statistical analysis using Minitab’s two-sample *t*-test consistently showed statistically significant differences (*P* < 0.05) among all the groups. Additionally, effect size analysis based on ANOVA and Cohen’s guidelines confirmed large effect sizes across the ESPs, indicating that the observed differences were statistically significant and practically meaningful. To address the potential inflation of type I errors due to multiple comparisons, the Bonferroni correction was applied to adjust the significance threshold, further validating the meaningful differences observed between the ESP groups. The Duoblade consistently produced the lowest number of additional smoke particles, with further reduction when the integrated suction was activated. All presented values represent additional particle generation above the baseline mean of 33 015 particles/cubic foot for 0.3-µm particles and 5454 particles/cubic foot for 0.5-µm particles (measured without ESP activation).

**Figure 2.**
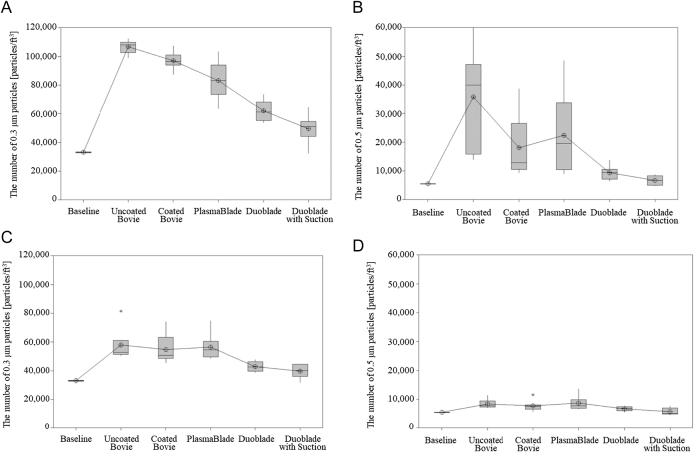
Quantitative evaluation of 0.3- and 0.5-μm particles in surgical smoke. (a) Number of 0.3 μm particles in 35 W cut mode, (b) number of 0.5 μm particles in 35 W cut mode, (c) number of 0.3 μm particles in 35 W coagulation mode, and (d) number of 0.5 μm particles in 35 W coagulation mode. *: Outlier, defined as a data point >1.5 times the interquartile range from the edge of the box.

In the cut mode, the effect size at 0.3 µm was 0.875, indicating a statistically significant difference between the groups according to Cohen’s guidelines. Statistical results are reported as means with 95% confidence intervals (CIs). Particle generation was the highest with the uncoated Bovie (mean: 73 569 particles/cubic foot, 95% CI: 67 007–80 130). Duoblade exhibited the lowest level of particle generation among all ESPs tested, satisfying the Bonferroni-adjusted significance threshold (α = 0.005 for five ESP groups), confirming that Duoblade demonstrated the most effective reduction in 0.3-µm particle generation in terms of both statistical significance and effect size. Duoblades without suction significantly reduced this to a mean of 28 998 particles/cubic foot (95% CI: 22 860–35 135), representing a 60.6% improvement over uncoated Bovie. The Duoblade with suction further enhanced the device’s low particle generation performance, achieving a mean of 16 321 particles/cubic foot (95% CI: 10 184–22 459), a 77.8% reduction compared with the uncoated Bovie, and a 43.7% reduction compared with the Duoblade without suction. Coated Bovie produced a mean of 63 949 particles/cubic foot (95% CI; 57 811–70 086), and PlasmaBlade produced a mean of 50 167 particles/cubic foot (95% CI: 44 030–56 304) in the cut mode (Fig. 2a).

In coagulation mode, the effect size at 0.3 µm was 0.501, indicating a statistically meaningful difference between groups according to Cohen’s guidelines, and overall particle generation at this size was lower than in cut mode (Fig. 2b). Uncoated Bovie produced a mean of 24 786 particles/cubic foot (95% CI: 19 040–30 532), coated Bovie produced a mean of 21 713 particles/cubic foot (95% CI: 15 967–27 460), and PlasmaBlade produced a mean of 23 368 particles/cubic foot (95% CI: 17 622–29 114). Similar to the findings in cut mode, Duoblade exhibited the lowest level of particle generation among all ESPs tested in coagulation mode, satisfying the Bonferroni-adjusted significance threshold (α = 0.005 for five ESP groups). This confirms that Duoblade consistently demonstrated the most effective reduction in 0.3-µm particle generation regarding statistical significance and effect size. Duoblades without suction emitted a mean of 9788 particles/cubic foot (95% CI: 4042–15 535), which is a 60.5% improvement over the uncoated Bovie. Activating the Duoblade’s suction further lowered this to a mean of 6702 particles/cubic foot (95% CI: 956–12 488), representing a 73.0% reduction compared with the uncoated Bovie and a 31.5% decrease compared with the Duoblade without suction.

For 0.5-µm particles in cut mode, the effect size was 0.39, indicating a statistically meaningful difference between groups according to Cohen’s guidelines. Among the tested ESPs, the uncoated Bovie showed the highest generation (mean: 25 818 particles/cubic foot, 95% CI: 17 245–34 392; Fig. 2c). Coated Bovie and PlasmaBlade generated means of 12 566 (95% CI: 3954–21 101) and 16 957 particles/cubic foot (95% CI: 8345–24 497), respectively. Duoblades without suction dramatically reduced this to a mean of 3914 particles/cubic foot (95% CI upper: 12 499), an improvement of 87.1%. The Duoblade with suction yielded the lowest emissions at a mean of 1176 particles/cubic foot (95% CI upper: 9711), a 96.1% reduction compared to the uncoated Bovie, and a 69.9% reduction compared to the Duoblade without suction. Because of the lower mean value observed for the Duoblade compared to the other ESPs, the lower bound of the calculated CI occasionally yielded a negative value. However, because particle generation must be positive, only the upper bound of the CI has been reported in such cases. At the conventional statistical significance level (*P* < 0.05), Duoblade demonstrated a significantly lower generation of 0.5-µm particles compared to other ESPs. However, this significance was not retained under the Bonferroni-adjusted threshold. This limitation may be attributed to the lower overall particle counts for 0.5-µm particles compared to the 0.3-µm case across all ESP groups, combined with the moderate sample size (*n* = 16), which may reduce statistical power. Although the Bonferroni-adjusted significance threshold was not met, the results suggest that the Duoblade effectively reduced particle generation and may offer advantages in removing larger particles through its integrated suction system during the cut mode.

Finally, 0.5-µm particle generation in coagulation mode was substantially lower across all devices compared with cut mode (Fig. 2d). However, the effect size was 0.339, indicating a statistically significant difference between the groups according to Cohen’s guidelines. Uncoated Bovie generated a mean of 2891 particles/cubic foot (95% CI: 1707–3998), coated Bovie generated a mean of 2282 particles/cubic foot (95% CI: 1099–3389), and PlasmaBlade generated a mean of 3205 particles/cubic foot (95% CI: 2022–4312). Although no statistically significant differences were found between the uncoated and coated Bovies, the other groups exhibited statistically significant differences under the general threshold (*P* < 0.05). As in the previous analyses, the differences between the Duoblade and other ESPs did not reach statistical significance under the more conservative Bonferroni-adjusted threshold. However, the results suggested that the Duoblade consistently demonstrated the lowest level of particle generation among the devices tested. Duoblades without suction generated a mean of 1266 particles/cubic foot (95% CI: 82–2372), a 56.2% improvement compared to the uncoated Bovie. With suction, the Duoblade further reduced this to a mean of 315 particles/cubic foot (95% CI upper: 1422), an 89.1% reduction compared to the uncoated Bovie, and an impressive 75.1% decrease compared to the Duoblade without suction. Previously, the negative lower confidence limit for Duoblade with suction was excluded because the particle counts should be positive.

The overall lower smoke generation in coagulation mode versus cut mode is attributable to the differences in the electrosurgical unit generator waveforms. Both the Medtronic AEX Generator (for PlasmaBlade) and the ValleyLab FT10 Generator (for other devices) employ higher voltages and pulsed waveforms in the coagulation mode, as detailed in their manuals. The ValleyLab FT10 generator was operated at a frequency of 434 kHz and generated a sinusoidal waveform. In the cut mode, it delivers a continuous waveform at a peak voltage of 700 V, whereas in the COAG mode, it produces a damped sinusoidal burst waveform at the 2300 V peak with a duty cycle of 6.25%. Conversely, the Medtronic AEX generator, which was used exclusively with PlasmaBlade, operated at 469 kHz and generated a sinusoidal waveform. In the cut mode, it delivers a continuous waveform at the 550 V peak, and in the COAG mode, it produces a damped sinusoidal burst waveform at the 2400 V peak with a 16% duty cycle. These pulsed waveforms facilitate effective coagulation via spark generation and deliver a lower average amount of energy to the tissue than the continuous waveforms used in the cut mode, resulting in reduced smoke generation.

### Blood loss

**Figure 3.**
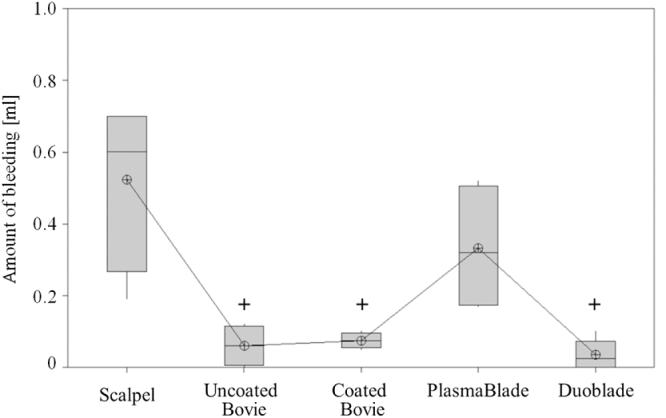
Quantitative evaluation of integrated blood loss following each incision. +: *P* < 0.05 versus scalpel.

Intraoperative blood loss varied among the surgical instruments, with measurements converted from grams to milliliters (1 g = 1 mL) for comparison (Fig. 3). An effect size of 0.73 confirmed that there were statistically significant differences in blood loss among the groups. The scalpel, which served as the control, showed a mean blood loss of 0.52 mL (95% CI: 0.39–0.65). However, differences in hemostatic efficacy were observed among the ESP devices. Specifically, the uncoated Bovie resulted in a mean blood loss of 0.06 mL (95% CI upper: 0.19), and the coated Bovie resulted in a mean blood loss of 0.075 mL (95% CI upper: 0.21). PlasmaBlade exhibited a higher mean blood loss of 0.33 mL (95% CI: 0.20–0.46), while Duoblade demonstrated the lowest mean blood loss of 0.035 mL (95% CI upper: 0.13), representing an average reduction in blood loss of approximately 93.3% compared with the scalpel. Duoblade showed a statistically significant difference compared with the scalpel and PlasmaBlade, satisfying the Bonferroni-adjusted significance threshold (α = 0.005 for five ESP groups).

### Histological analysis

**Figure 4.**
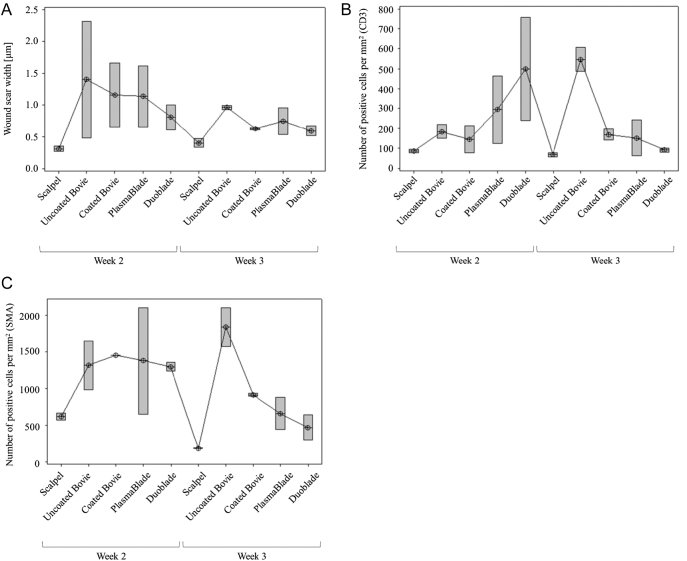
Histological assessment of wound healing, inflammation, and fibrosis at incision sites. (a) Quantitative analysis of the wound scar width by MT staining. (b) CD3-positive T-cell infiltration at incision sites. (c) α-SMA-positive myofibroblasts for fibrotic tissue remodeling.

**Figure 5.**
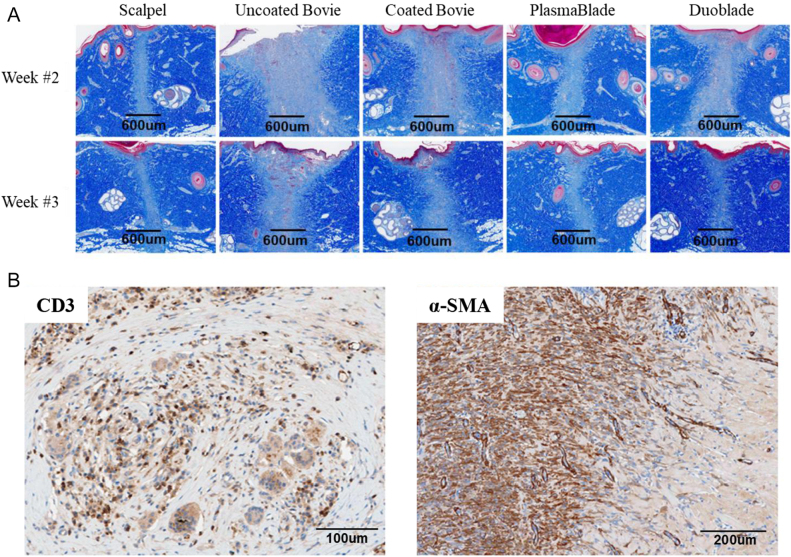
Histological images. (a) Representative images depicting the wound scar width of incisions made by each instrument (MT staining, ×3.5). The wound scar width of incisions made with Duoblade was similar to or wider than that of incisions made with the other instruments at week 2, but became the second narrowest by week 3. (b) Representative images of CD3 and α-SMA IHC staining. The numbers of CD3- and α-SMA-positive cells per mm^2^ were assessed in the wound scar area. a, ×21.7; b, ×12.9.

The wound scar width was the narrowest following scalpel incisions at both the second and third weeks. The scars produced by the Duoblade were narrower than those produced by any other instrument. In CD3- and α-SMA-stained slide, the mean size of the selected regions was 5.02 (range: 1.47–10.10) and 4.61 mm^2^ (range: 1.16–16.25), respectively. One-week samples did not exhibit a definable wound-healing architecture, and re-epithelialization and granulation tissue formation were minimal or absent, making meaningful histological comparisons between ESPs infeasible. As this study aims to evaluate differences in the inflammatory response and fibrosis, which typically emerge after the early inflammatory phase, formal analysis excluded the 1-week data. Therefore, intermediate time points (2 and 3 weeks) were selected to allow for a reliable evaluation of wound remodeling and fibrosis progression. The density of CD3-positive inflammatory cells was the lowest in scalpel-incised wounds (mean: 85.4/mm^2^ at week 2 and 67.1/mm^2^ at week 3). The CD3-positive cell density in Duoblade specimens peaked at week 2 (mean: 498.2/mm^2^) but declined sharply by week 3, falling below the levels seen with the other three instruments (mean: 546.1, 168.2, and 150.3/mm^2^ for uncoated Bovie, coated Bovie, and PlasmaBlade, respectively). Likewise, myofibroblast density in the scar was the lowest in scalpel-incised wounds. At week 2, the myofibroblast density in the Duoblade specimens was comparable to that in the other instrument groups (mean: 1313.8, 1447.6, 1375.0, and 1291.6/mm^2^ for the uncoated Bovie, coated Bovie, PlasmaBlade, and Duoblade, respectively). By week 3, it was the second lowest among the four ESPs (mean: 1837.4, 908.8, 655.9, and 465.0/mm^2^ for the uncoated Bovie, coated Bovie, PlasmaBlade, and Duoblade, respectively; Figs 4 and 5).

MT-stained sections show progression of wound healing using different surgical instruments at weeks 2 and 3. At both time points, the narrowest scar widths were observed in the scalpel-incised group (mean: 0.311 µm at week 2 and 0.402 µm at week 3), with minimal disruption to the surrounding dermal tissue. Wounds generated by Duoblade demonstrated narrower scar regions (mean: 0.809 µm at week 2 and 0.594 µm at week 3) than those created by the uncoated Bovie (mean: 1.40 µm at week 2 and 0.962 µm at week 3), coated Bovie (mean: 1.16 µm at week 2 and 0.629 µm at week 3), and PlasmaBlade (mean: 1.14 µm at week 2 and 0.747 µm at week 3).

Histological differences between ESPs were statistically more pronounced at week 3, with effect sizes for the CD3-positive cell density, myofibroblast density, and scar width of 0.92, 0.91, and 0.76, respectively. Excluding scar width, all comparisons reached statistical significance at the conventional threshold of *P* < 0.05. Owing to rapid growth during the experimental period, frequent wound dehiscence in the back area by week 4 limited the ability to collect reliable long-term healing data. However, this analysis illustrated that scalpel incisions resulted in the most limited scar formation, and Duoblade incisions were associated with reduced scar width and a more confined fibrotic response at later time points.

## Discussion

This study aims to quantitatively evaluate the performance of various ESPs, focusing on surgical smoke production and thermal tissue damage. Our findings demonstrate that the design and energy delivery mechanisms of ESPs significantly influence both environmental and biological outcomes during surgery.

One of the most noteworthy findings of this study was the substantial variation in surgical smoke generation among the tested ESPs. The uncoated Bovie, a conventional high-temperature dissection device, produced the greatest number of airborne particles, especially in the cut mode, consistent with its exposed metal electrode design. In contrast, the Duoblade, which operates via dielectric heating and features a fully insulated tip, generates significantly fewer smoke particles. The use of integrated suction with the Duoblade further enhanced this reduction, lowering the particle count by up to 96.1% compared to the uncoated Bovie under certain conditions.

These findings are consistent with prior literature suggesting that dielectric-based and low-temperature devices produce fewer thermal by-products and thus generate less surgical smoke^[^32^]^. Moreover, the efficacy of local smoke evacuation, particularly if integrated into the ESP itself, aligns with the consensus on minimizing occupational exposure to surgical plumes, as supported by existing research^[^13^]^.

Although low-temperature ESPs are often thought to sacrifice hemostatic performance to reduce thermal injury, our results challenge this notion. The Duoblade demonstrated a hemostatic capability superior to that of all other devices, including high-temperature ESPs, with blood loss reduced by more than 93% compared to that of the scalpel. This suggests that the dielectric heating method used by the Duoblade effectively coagulates tissues without excessive thermal spread, which is a critical factor in minimizing blood loss and maintaining surgical field visibility. Notably, PlasmaBlade, another low-temperature ESP, showed inferior hemostatic control, with blood loss closer to that of the scalpel. The lower hemostatic capability of the PlasmaBlade is attributable to its operating characteristics, which may limit effective vascular coagulation. Contrastingly, the Duoblade achieved a hemostatic performance comparable to or superior to that of high-temperature dissection ESPs, such as uncoated and coated Bovies. The hemostatic effect of the Duoblade is likely attributable to its tissue dielectric heating mechanism and specific electrode design. This highlights that low-temperature technologies are not equivalent in terms of performance and that device-specific design features, such as waveform modulation and electrode geometry, critically influence the outcomes.

Histological analysis further substantiated the advantages of the Duoblade. Although scalpel incisions showed the least inflammation and fibrosis, owing to the absence of thermal injury, Duoblade incisions demonstrated the narrowest scar widths among the ESP groups. The densities of CD3-positive lymphocytes and α-SMA-positive myofibroblasts were both initially high at week 2, but declined significantly by week 3, indicative of rapid resolution of the inflammatory response and a lower risk of long-term fibrotic remodeling. These histological findings suggest that the Duoblade achieves an optimal balance between effective tissue dissection and minimal collateral damage. Compared to PlasmaBlade, which showed a slower resolution of inflammation, Duoblade appeared to support more favorable wound-healing trajectories.

This study compared FDA 510(k)-cleared ESPs regarding smoke generation, tissue effects, and wound-healing outcomes. Devices, such as PlasmaBlade, require proprietary generators, posing cost and compatibility barriers, whereas others, such as the Duoblade, are compatible with standard three-pin electrosurgical units, improving their clinical accessibility. Importantly, the Duoblade demonstrated the lowest smoke production and tissue recovery. Surgeons have the right to be informed about how different ESPs affect surgical smoke and tissue integrity, and this evidence may help guide more informed and safer device selection in clinical practice.

This study has several limitations. Although the porcine skin model closely mimics human dermal properties, extrapolation of the results to internal organ surgery requires caution. Future studies should include long-term follow-up and assessment of more clinically diverse tissue types. Additionally, further investigation of the energy profiles and real-time thermal dynamics of these devices would help clarify the mechanistic basis of their different performances. The evaluation of user ergonomics, cost-effectiveness, and patient-centered outcomes would also be valuable for clinical adoption.

## Conclusion

This study demonstrated that the Duoblade electrosurgical unit, which uses dielectric heating and integrated suction, significantly reduces surgical smoke and tissue damage while maintaining excellent hemostatic performance. Compared to conventional ESPs, Duoblade showed superior environmental safety and wound healing outcomes. These findings highlight its potential as a safer and more effective surgical tool in modern operating rooms.

## Data Availability

The data are available upon reasonable request to the corresponding author.

## Research registration unique identifying number (UIN)

This is an animal study.
